# Microbiome and Allergic Diseases

**DOI:** 10.3389/fimmu.2018.01584

**Published:** 2018-07-17

**Authors:** Mariona Pascal, Marina Perez-Gordo, Teresa Caballero, Maria M. Escribese, M. Natividad Lopez Longo, Olga Luengo, Luis Manso, Victor Matheu, Elena Seoane, Miguel Zamorano, Moisés Labrador, Cristobalina Mayorga

**Affiliations:** ^1^Immunology Department, Centro de Diagnóstico Biomédico, Hospital Clínic de Barcelona, Institut d’Investigacions Biomèdiques August Pi i Sunyer (IDIBAPS), Universitat de Barcelona, ARADyAL, Barcelona, Spain; ^2^Basic Medical Science Department, Faculty of Medicine, CEU San Pablo University, ARADyAL, Madrid, Spain; ^3^Institute of Applied and Molecular Medicine (IMMA), Faculty of Medicine, CEU San Pablo University, Madrid, Spain; ^4^Hospital Universitario La Paz, Madrid, Spain; ^5^Hospital Universitario de Araba, Vitoria, Spain; ^6^Hospital Vall d’Hebron, Barcelona, Spain; ^7^Hospital Universitario del Sureste, Madrid, Spain; ^8^Hospital Universitario de Canarias, Santa Cruz de Tenerife, Spain; ^9^Hospital Universitario Gregorio Marañón, Madrid, Spain; ^10^Hospital Universitario Ramón y Cajal, Madrid, Spain; ^11^Research Laboratory and Allergy Unit, Instituto de Investigación Biomédica de Málaga (IBIMA), Hospital Regional Universitario, Universidad de Málaga, ARADyAL, Malaga, Spain

**Keywords:** microbiome, microbiota, allergy, allergic diseases, prebiotics, probiotics, synbiotics

## Abstract

Allergic diseases, such as respiratory, cutaneous, and food allergy, have dramatically increased in prevalence over the last few decades. Recent research points to a central role of the microbiome, which is highly influenced by multiple environmental and dietary factors. It is well established that the microbiome can modulate the immune response, from cellular development to organ and tissue formation exerting its effects through multiple interactions with both the innate and acquired branches of the immune system. It has been described at some extent changes in environment and nutrition produce dysbiosis in the gut but also in the skin, and lung microbiome, inducing qualitative and quantitative changes in composition and metabolic activity. Here, we review the potential role of the skin, respiratory, and gastrointestinal tract (GIT) microbiomes in allergic diseases. In the GIT, the microbiome has been proven to be important in developing either effector or tolerant responses to different antigens by balancing the activities of Th1 and Th2 cells. In the lung, the microbiome may play a role in driving asthma endotype polarization, by adjusting the balance between Th2 and Th17 patterns. Bacterial dysbiosis is associated with chronic inflammatory disorders of the skin, such as atopic dermatitis and psoriasis. Thus, the microbiome can be considered a therapeutical target for treating inflammatory diseases, such as allergy. Despite some limitations, interventions with probiotics, prebiotics, and/or synbiotics seem promising for the development of a preventive therapy by restoring altered microbiome functionality, or as an adjuvant in specific immunotherapy.

## Introduction

Allergic diseases, include heterogeneous inflammatory pathologies such as respiratory and food allergies (FA), which are characterized by an immunological response with T lymphocytes producing IL-4, IL-5, and IL-13 and low production of IFN-γ (Th2) (1) and others producing IL-9 and IL-10 (Th9) (2) as the main effector T cells. They promote the induction of other effector cells involved in allergic inflammation, such as mast cells, basophils, and eosinophils (1). These diseases have dramatically increased in prevalence over the last few decades (3–6) and recent research points to a central role of the microbiota (7, 8). It is well established that the microbiome can modulate the immune response, from cellular development to organ and tissue formation (9) exerting its effects through multiple interactions with both the innate and acquired branches of the immune system. In the late 80s, Dr. Strachan proposed what is now referred to as the “hygiene-hypothesis” (10), in which changes in environment and nutrition produce a dysbiosis in the skin, gut, or lung microbiome inducing qualitative and quantitative changes in composition and metabolic activity (11, 12). Furthermore, it was proposed that a lower incidence of infection in early childhood, which may be associated with low microbiota diversity, could explain the increase in prevalence of atopic diseases (13). It should be pointed out that the hygiene hypothesis has not been found to apply to individual hygiene [no relation between personal or home cleanliness and increased risk of asthma or allergy has been found (14)], but to independent host factors such as number of older siblings, contact with pets and rural versus urban living, all of which have been shown to affect microbiome composition and the development of immunologic tolerance (15). Today, the use of bacterial culture-independent tools such as next-generation sequencing to identify different microbes has permitted the investigation of complex populations and their roles in health and disease. Here, we review the potential role of the skin, respiratory, and gastrointestinal tract (GIT) microbiomes in allergic diseases.

## Microbiome

The term “microbiome” refers to the microorganisms that live on or inside another organism. They interact with each other and with their host and can be classified as beneficial (symbiotic) or dangerous (pathogenic) (16). Microbiome in humans can account for 90% of the cells by a ratio of 10:1 (17). New studies point out that the number of bacteria in the body is of the same order as the number of human cells (18). Most of these microorganisms inhabit the gut. The microbiome effectively adds a huge amount of genes to the human genome, potentially increasing it up to 200 times (19). As a result, the composition of the human microbiome could be important in the context of health or disease.

### Human Gut Microbiome and Implications in Food Allergy

The GIT has a very important immune function in developing either effector or tolerant responses to different antigens by balancing the activities of Th1 and Th2 cells as well as regulating Th17 and T regulatory (Treg) cells in the lamina propria (20–23). Immune dysfunction in allergic diseases such as asthma and atopy seems to be related to differences in the function and composition of the gut microbiome (24).

The gut microbiome constitutes a highly complex ecosystem which includes eukaryotic fungi, viruses, and some archaea, although bacteria are the most prominent components (25). Its composition is generally formed during the first 3 years of life (26); however, recent work has suggested that its colonization may begin *in utero* (27), contrary to the widely held dogma of the fetus as a sterile environment. Despite its early formation, its composition is highly dynamic and dependent on host-associated factors such as age, diet, and environmental conditions (26, 28–31) with the major phyla being *Actinobacteria, Bacteroidetes, Firmicutes*, and *Proteobacteria*. The gut microbiome is not homogeneous throughout the GIT, showing higher diversity in the oral cavity and intestine, and lower diversity in the stomach, mainly because of the acid environment (32). Aerobic species are mainly located in the upper small intestine and anaerobic species in the colon (33).

**Figure 1:**
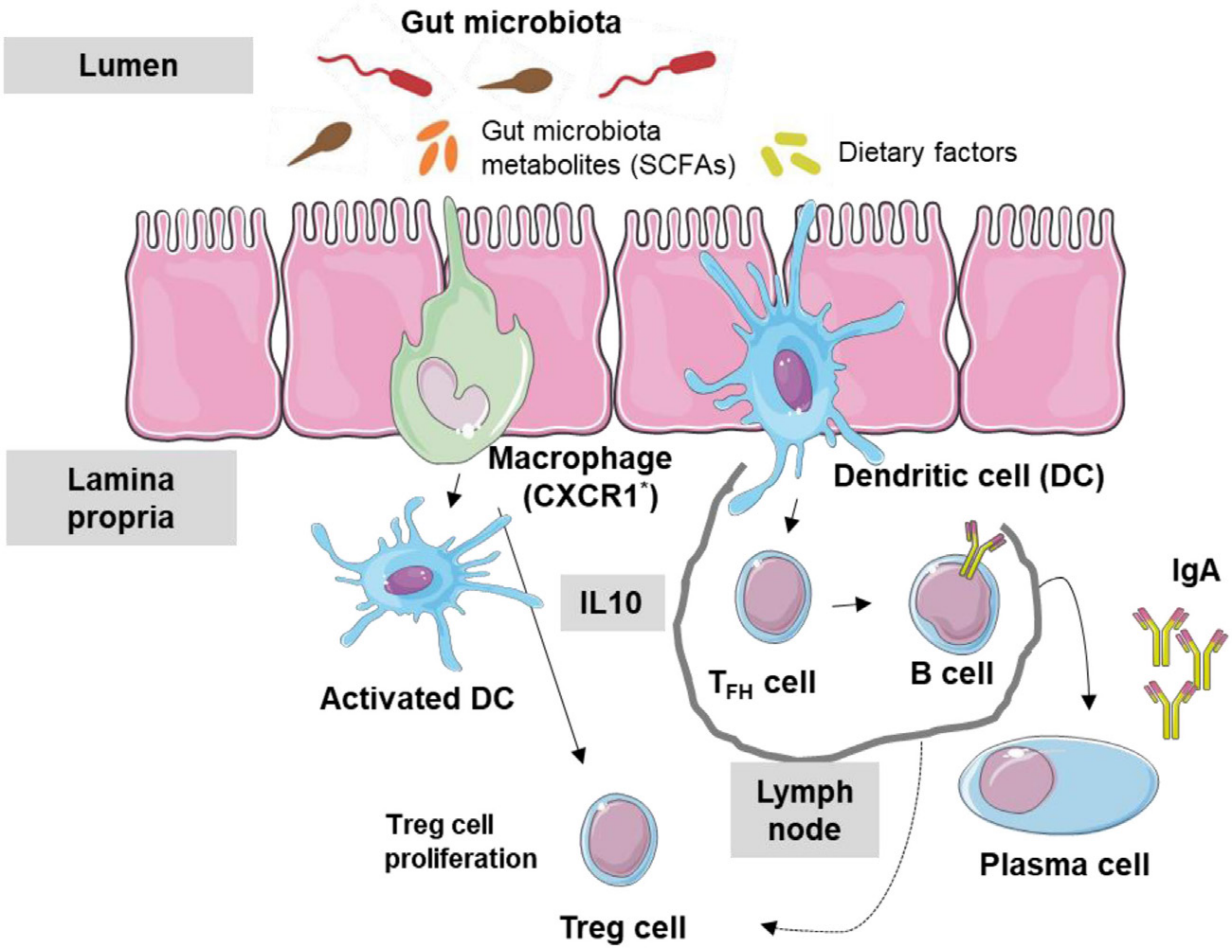
Interaction between gut microbiota and immune system. Gut microbiota metabolites and dietary factors constitute the main antigen load of the gastrointestinal tract. Macrophages (CXCR1^+^) and dendritic cells (DCs) are stimulated and T regulatory (Treg) cells are activated by metabolic products such as short chain fatty acid (SCFA). Follicular T cells activate B cells inducing the production of IgA antibodies.

Most antigens in the GIT come from dietary factors and gut microbiota, both of which can affect immune tolerance being the promotion of Treg cells to these dietary factors crucial to avoid an immune response to dietary antigens (34). Alterations in GIT bacterial levels or diversity (dysbiosis) can disrupt mucosal immunological tolerance, leading to allergic diseases including FA (35) and even asthma (36–38). Moreover, low IgA levels at the intestinal surface barrier can also contribute to FA. In fact, low microbiota levels and IgA appear to be related: gut microbiota can stimulate dendritic cells (DCs) in the Peyer’s patches (digestive type of mucosa lymphoid-associated tissue) to activate B cells, leading to specific IgA antibodies production through class switching (39). This stimulation may occur through the production by members of the microbiome of metabolites, such as short chain fatty acids (SCFAs). Thus, the immune tolerance network in the intestinal lumen can be considered to include the gut microbiota, their metabolic products, dietary factors, epithelial cells, DCs, IgA antibodies, and regulatory T cells (Figure 1).

Several factors associated with dysbiosis may influence FA, such as cesarean versus vaginal delivery (40), low versus rich fiber diet (41), breastfeeding (42), and/or early-life-antibiotic exposure, all of which affect bacterial load and diversity.

Once thought to be almost sterile, the esophagus has been shown to comprise around 300 bacteria species. Significant differences in the microbial composition of children with active esophageal inflammation caused by eosinophilic esophagitis compared with controls have been reported (43). Importantly, both the degree of inflammation and the treatment regimen seem to impact the esophageal microbiota (43).

### Human Lung Microbiome and Implications in Respiratory Allergy

As with the esophagus and fetus, the lung has long been thought of as sterile; however, recent evidence has shown it to harbor various bacteria phyla, including *Actinobacteria, Bacteroidetes, Firmicutes*, and *Proteobacteria*, even in healthy subjects (44). Similar to the gut, the lung microbiome changes rapidly in the first years of life, before beginning to stabilize (45, 46). Colonization occurs gradually in healthy children, starting with *Staphylococcus* or *Corynebacterium*, followed by *Moraxella* or *Alloiococcus* (46). A breakdown in the development of the commensal population can lead to dysregulation of the IgE–basophil axis, with elevated serum IgE concentrations and increased of circulating basophil populations as has been described in murine models of allergic airway disease (47). Importantly, this link was found to be B-cell intrinsic and dependent on the MYD88 pathway. Moreover, the lung microbiome may also play a role in driving asthma endotype polarization, by adjusting the balance between Th2 and Th17 patterns. *Enterococcus faecalis* can suppress Th17 immunity and symptoms of allergic airway disease, and thus it has even been considered a potential therapeutic agent for both asthma and Th17 immunity (48).

Differences in levels and diversity of the lung microbiome have been found between healthy people and patients with asthma and allergic diseases, with an increase of Proteobacteria in the latter; moreover, their presence has been linked to increased severity of asthma probably through the upregulation of Th17-related genes (49, 50).

Early colonization with *Haemophilus influenzae, Moraxella catarrhalis*, and *Streptococcus pneumoniae* has been associated with recurrent wheezing and asthma (45, 46, 51, 52). Importantly, as well as bacteria, viruses will also influence asthma development, as has been demonstrated with human rhinovirus infections of the nasopharynx in early-life (46). In addition, other associations such as helminths may be protective for asthma, as helminth infections have been shown to increase the microbiota diversity (53). Associations have been found between the composition of the lung and gut microbiome and the risk of respiratory allergic disease development (54) indicating that both gut and lung mucosa may function as a single organ, sharing immunological functions (44).

### Skin Microbiome and Cutaneous Allergic Diseases

Bacterial dysbiosis is associated with chronic inflammatory disorders of the skin, such as atopic dermatitis (AD) and psoriasis (55). The composition of the skin microbiota depends on the body site samples (56). The relevance of AD, often associated with other allergic diseases, has significantly increased in the last few decades. Outgrowths of *Staphylococcus* and reductions of other communities like *Streptococcus* or *Propionibacterium* species correlate with AD flares (57). On the other hand, skin commensal *Acinetobacter* species have been reported to protect against allergic sensitization and inflammation, playing an important role in tuning the balance of Th1, Th2, and anti-inflammatory responses to environmental allergens (58). Interestingly, studies of cutaneous allergic diseases have found an association with gut microbiome dysbiosis (59), although the underlying mechanisms are still unclear. An initial study of 90 patients with established AD found enrichment for *Faecalibacterium prausnitzii* and decreased levels of SCFAs in the gut (60).

**Figure 2:**
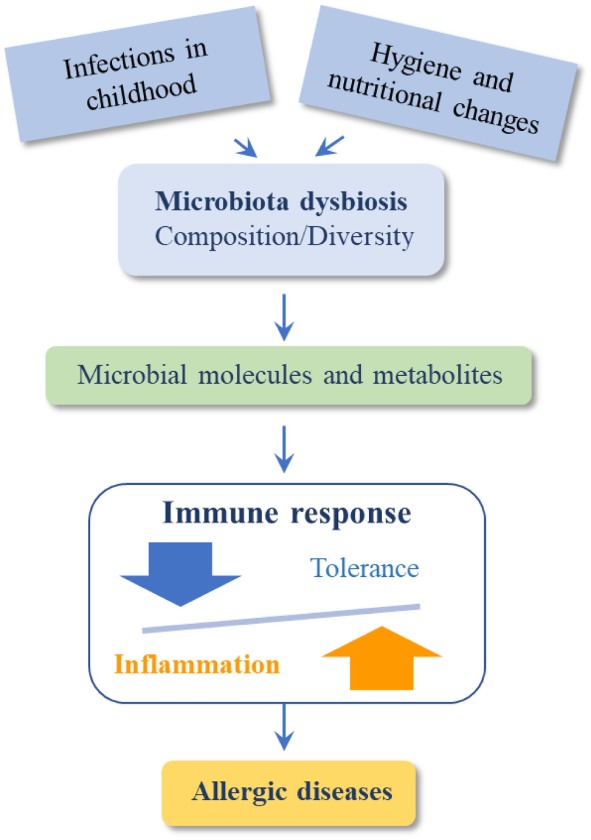
Dysbiosis induce qualitative and quantitative changes in the microbiota that directly affect immunological mechanisms leading to allergic diseases.

Therefore, we can summarize that changes in environment and diet produce dysbiosis in gut, skin, and/or lung microbiome inducing qualitative and quantitative changes in the microbiota which directly affect the immunological mechanisms implicated in the prevention of allergic diseases (Figure 2).

## Factors Affecting Microbiome Diversity

### Childbirth

The mode of delivery in childbirth can produce profound differences in the infant gut microbiome, with lower level of *Escherichia coli, Bifidobacterium*, and *Bacteroides* species in children born through cesarean section compared with those delivered vaginally (28, 61, 62). Cesarean-born infants typically have a microbiome enriched with *Staphylococcus* and *Streptococcus*, comparable with the maternal skin microbiome (63). These differences appear to be associated with higher risk of allergic diseases and asthma (64–66). Transfer of maternal vaginal microbes at birth may mitigate these effects (67). Time of gestation may also be a factor: premature births are associated with alterations of the gut microbiome, but not atopic sensitization (68).

### Importance of Early-Life Microbiome

There is mounting evidence that early-life exposure is critical for the microbiome and that gut microbial dysbiosis heavily influences immune system development (53). Potential factors include perinatal exposure to maternal or infant diet, antibiotic use, and contact with older siblings (16). Data from different populations show that the highest interindividual microbial variability occurs during the first 3 years of age (26). Noteworthy, contact with the microbiome can start before birth, since a low-abundance microbiota in the placenta (69) and meconium (70, 71) have been found.

Microbial exposure during the first months of life induces the activation of the innate immune system in different ways, with consequences for FA. Early inoculation with spore-forming *Clostridium* class IV and XIV species (72) and other bacteria (53) leads to decreased levels of circulating IgE in adulthood. Conversely, 3-week-old neonates with a higher fecal burden of *Clostridium difficile* and a higher ratio of *C. difficile* to *Bifidobacterium* showed increased numbers of skin test positive results to food and aero-allergens (73). Similarly, high levels of fecal *E. coli* in infants during their first month are associated with IgE-mediated eczema (74, 75).

Remarkably, the same colonization pattern can have different consequences at different ages. For example, colonization of *S. pneumoniae, H. influenzae*, or *M. catarrhalis* within the first month of life increases the risk of asthma, leading to high counts of atopic markers such as eosinophils and serum IgE, but not when colonization occurs at 12 months (45).

Furthermore, respiratory tract infections during early-life are associated with asthma development (76, 77). This may be because viral infections favor other opportunistic respiratory pathogens such as *M. catarrhalis* and *S. pneumoniae*, increasing the risk of asthma exacerbations (78). Other possible mechanisms may involve respiratory rhinovirus interacting with airway epithelial cells, increasing IL-25 and IL-33 production and contributing to Th2 immune responses (79). This is in line with the higher levels of house dust mite-specific IgE found in children infected with rhinovirus (80). Moreover, rhinovirus infection can also induce mucus hypersecretion and airway hyperresponsiveness in neonatal mice compared with adults (81).

### Diet and Microbiome Metabolic Products

Another key factor influencing gut microbiome diversity is infant feeding, and especially breastfeeding, which has been shown to increase colonization by *Lactobacilli* and *Bifidobacteria* (82). Breast milk contains oligosaccharides and a wide range of fatty acids, which will affect the gut microbiome and its capacity to produce metabolites that protect against allergies and asthma (83) through the development of Treg cells (84). This effect is also produced by the intake of unprocessed milk during the first year of life, probably related to higher levels of peptides in the serum fraction and unsaturated omega-3 fatty acids (85). Other dietary components such as polyphenols and fish oils are also important for microbiome diversity (86–88).

Some noteworthy bacteria, such as *Lachnospiraceae* and *Ruminococcaceae*, can also influence the gut microbiome by producing SCFAs—including propionate, butyrate, and acetate—through fermentation of complex dietary carbohydrates. Importantly, besides acting as an essential energy source for gastrointestinal colonocytes, these acids exert various anti-inflammatory effects on the immune system that can modulate FA and respiratory diseases (89, 90), by increasing epithelial barrier function (91), and inducing Treg cells (colonic CD103^+^FoxP3^+^ cells), DCs precursors, and IL-10 production (8, 90).

### Importance of Exposure to Antibiotics

The introduction of antibiotics in the 1950s is associated with an increasing incidence of allergy. This is thought to be causes by antibiotics inducing dysbiosis which has been shown to directly impact the development of AD (92) and asthma (48). The age of initial exposure could be important since maternal intake of antibiotic during pregnancy increases the risk of allergy in children (93), and antibiotic use in the first month of life has been associated with cow’s milk allergy (94). Intrapartum antibiotics have been shown to lead to a modified microbiome in children at 3 and 12 months (95). Other studies showed that antibiotics affect the microbiome in older subjects (96, 97). Antibiotic administration is associated with severe allergic airway inflammation in neonates, but not in adults (98).

Even low doses of antibiotics can affect microbiome composition (99); however, the associations between antibiotic consumption and allergic diseases increase with the number of antibiotics prescribed, and variable effects have been found for different antibiotic families. Some studies have indicated that betalactam antibiotics are the most common triggers when FA is diagnosed before 2 years of age, while macrolides are associated with FA when it is diagnosed later (100). For asthma, further studies are needed to clarify whether it is the infection rather than the antibiotics themselves that increase susceptibility (101).

## Interventions

The microbiota can be considered a therapeutical target for treating allergy; moreover, certain species can be used to enhance tolerance response induction. Different approaches for restoring the microbiome involve probiotics, prebiotics, and synbiotics.

### Probiotics

According to the Food and Agriculture Organization of the United Nations and the World Health Organization, probiotics are defined as “*live microorganisms which, when administered in adequate amounts, confer a health benefit to the host*” (102). They do so by promoting the appropriate balance of gut microbiota. The health benefits attributed to one probiotic strain are not necessarily applicable to another one even within one given species (103). Furthermore, the effectiveness may depend on the time of intervention and aspects of the current microbiota composition. In fact, different studies have shown that timing is crucial (104).

In the case of FA, co-administration of bacterial adjuvants with oral immunotherapy (OIT) has been suggested as a potential treatment. Probiotic therapy with *Lactobacillus rhamnosus* increases efficacy when co-administered with peanut OIT—producing desensitization in 82% of treated patients (105)—or with hydrolyzed casein in milk allergic patients, in which an increase of fecal butyrate levels were found (106, 107). However, other strains of *Lactobacilli* and/or *Bifidobacteria* did not demonstrate any effect in preventing allergic diseases (106, 107). Some investigations have shown that the oral administration of probiotics may benefit allergic rhinitis patients (108–110); similarly, local nasal administration of *Lactococcus lactis* NZ9000 can affect local and systemic immune responses against *S. pneumoniae* (111). However, Ivory et al. reported that even oral delivery of *Lactobacillus casei* Shirota modified the immune system of allergic individuals (110), these modifications did not have a significant impact on the allergic status (112), highlighting the fact that analysis of immune parameters *per se* is not a real indicator of the therapeutical properties of the probiotics.

It has been suggested that probiotics can help preventing eczema and they also show some beneficial effects for other allergic diseases including asthma (113–117); furthermore, another approach based on the intranasal application of bacterial products (endotoxin or flagellin) has demonstrated immunomodulatory ability, mimicking the effect of probiotics, for the lung in different animal models, reducing experimental asthma by either re-establishing the expression of the ubiquitin-modifying enzyme A20 at the endothelial barrier or inducing Tregs (118, 119).

Therefore, it seems that the optimal time periods to apply probiotic intervention are before, during, and just after birth represents. Nevertheless, more studies, using clinical trial methodologies when possible, should be carried out to confirm these findings and determine the optimal probiotics to use.

### Prebiotics

Prebiotics are non-digestible food components that benefit the host by selectively stimulating the growth and activity of microorganisms. Studies have shown that fibers and oligosaccharides can improve immunity and metabolism (8) and that the treatment of pregnant and lactating mice increases the proportions of *Lactobacillus* and *Clostridium leptum* and promotes a long-term protective effect against FA in the offspring (120).

Studies evaluating the effect of fiber/oligosaccharide intake in modulating asthma (121–123) have shown heterogeneous results, with one study reporting a reduction of wheezing (121) but others reporting no effect (122, 123). A recent Cochrane review has shown that although the addition of prebiotics to infant food may reduce the risk of eczema, it is not clear whether their use may affect other allergic diseases including asthma (124).

### Synbiotics

When the use of a combination of prebiotics and probiotics produce synergistic health benefits it is described as a symbiotic. In FA mice models, both the microbiome and diet can affect the development of food tolerance by the induction of Treg cells (34). In cow’s milk allergy, it has been demonstrated that treatment with extensively hydrolyzed casein formula plus *L. rhamnosus* GG promotes tolerance through changes in the infant gut microbiome (89).

A recent meta-analysis has shown their beneficial effects for eczema treatment (125). However, further well-conducted, randomized, placebo-controlled longitudinal studies are still needed in this area (126).

## Conclusion

The microbiota is a highly dynamic environment influenced by multiple environmental and dietary factors, with a complex role in allergic diseases. Further studies with larger number of well-characterized patients and controls are needed to dissect the role of microbiome in allergic diseases are the performance. Despite some limitations, interventions with probiotics, prebiotics, and/or synbiotics show promise for the development of a preventive therapy, either by restoring altered microbiome functionality due to dysbiosis or as a boosting of immunological system in specific immunotherapy. However, the field is still relatively new and we expect many key findings to be made in the next few years. Detailed prospective, randomized, placebo-controlled studies will be essential for this purpose.

## Author’s Note

All authors belongs to the Immunology Committee of the Spanish Society of Allergy and Clinic Immunology (SEAIC).

## Author Contributions

CM, MP and MP-G conceived and designed this manuscript and were involved in manuscript production contributing equally to this work. TC, MME, MNLL, OL, LM, VM, ES, MZ and ML have read, revised and approved the manuscript.

## Conflict of Interest Statement

The authors declare that the research was conducted in the absence of any commercial or financial relationships that could be construed as a potential conflict of interest.
